# A Lectin with Highly Potent Inhibitory Activity toward Breast Cancer Cells from Edible Tubers of *Dioscorea opposita* cv. Nagaimo

**DOI:** 10.1371/journal.pone.0054212

**Published:** 2013-01-21

**Authors:** Yau Sang Chan, Tzi Bun Ng

**Affiliations:** School of Biomedical Sciences, The Chinese University of Hong Kong, Hong Kong; Aligarh Muslim University, India

## Abstract

A 70-kDa galactose-specific lectin was purified from the tubers of *Dioscorea opposita* cv. nagaimo. The purification involved three chromatographic steps: anion exchange chromatography on a Q-Sepharose column, FPLC-anion exchange chromatography on a Mono Q column, and FPLC-gel filtration on a Superdex 75 column. The purified nagaimo lectin presented as a single 35-kDa band in reducing SDS-PAGE while it exhibited a 70-kDa single band in non-reducing SDS-PAGE suggesting its dimeric nature. Nagaimo lectin displayed moderate thermostability, retaining full hemagglutinating activity after heating up to 62°C for 30 minutes. It also manifested stability over a wide pH range from pH 2 to 13. Nagaimo lectin was a galactose-specific lectin, as evidenced by binding with galactose and galactose-containing sugars such as lactose and raffinose. The minimum concentration of galactose, lactose and raffinose required to exert an inhibitory effect on hemagglutinating activity of nagaimo lectin was 20 mM, 5 mM and 40 mM, respectively. Nagaimo lectin inhibited the growth of some cancer cell lines including breast cancer MCF7 cells, hepatoma HepG2 cells and nasopharyngeal carcinoma CNE2 cells, with IC_50_ values of 3.71 µM, 7.12 µM and 19.79 µM, respectively, after 24 hour treatment with nagaimo lectin. The induction of phosphatidylserine externalization and mitochondrial depolarization indicated that nagaimo lectin evoked apoptosis in MCF7 cells. However, the anti-proliferative activity of nagaimo lectin was not blocked by application of galactose, signifying that the activity was not related to the carbohydrate binding specificity of the lectin.

## Introduction

Lectins are a group of proteins or glycoproteins possessing carbohydrate binding capability. In the past, lectins are classified in accordance with their carbohydrate specificities: mannose binding [1], mannose and glucose binding [2], galactose binding [3], etc. As more lectins with diverse sugar-binding specificities were identified, the old system was no longer feasible, and other classification systems were proposed. Animal lectins are classified into families of evolutionary related carbohydrate recognition domains (CRDs). Some major families include calnexins [4], M-type, L-type, P-type, R-type, S-type [5], I-type [6] and C-type [7] lectins. On the other hand, Van Damme *et* *al*. introduced another classification system of lectins, based on the structure of lectins, in which were classified into merolectins, hololectins, chimerolectins and superlectins [8]. They have also classified plant lectins into 12 groups according to their structural and evolutionary relationships, such as legume lectins, jacalins, amaranthins [9], [10]. Both animal and plant lectins exhibit a variety of biological activities. Some of them exert immuno-modulatory activities [11], [12], while others elicit anti-tumor, anti-bacterial, anti-fungal, anti-viral and anti-insect effects [13], [14]. The physiological functions and mechanisms of various animal lectins have been studied precisely [15]–[18]. However, those of plant lectins have not been clarified. There are still numerous plant lectins yet to be identified and studied. Plant storage organs are rich in proteins. They are one of the best targets for identification and isolation of new plant lectins with a large yield, allowing extensive characterization of the lectins, helping to reveal their biological potentials (e.g. anti-tumor activities).

Yam encompasses a number of species in the genus *Dioscorea*. Some examples of the major members are *D. alata* (purple yam), *D.* *bulbifera* (air potato), *D. cayenensis* (yellow yam), *D. dumetorum*, (bitter yam), *D. rotundata* (white yam), *D. opposita* (Chinese yam) and *D.* *trifida* (cush-cush yam). They are characterized by the possession of edible tubers that are rich in starch, some ions and vitamins. However, most of the yam tubers have to be cooked before consumption, in order to remove the toxic compounds present [19], [20].

Nagaimo is a Japanese mountain yam, which is a cultivar of *Dioscorea opposita*. Unlike tubers from other yams, *D. opposita* tubers are generally non-toxic that can be eaten raw after neutralization of oxalate crystals on the tuber skins. *D. opposita* tubers are used in many Japanese cuisines. Some of the tubers were utilized in traditional Chinese medicine, revealing their therapeutic potentials [21]. Although hemagglutinating activity was found in some types of yam tubers, there were very few investigations on the yam lectins. Also, none of the studies involving *D. opposita* tubers focused on the lectin. In a preliminary experiment, we detected the presence of a lectin in nagaimo (*D. opposita*) tubers that have not been examined before. We have therefore isolated the lectin, and studied its properties as well as biological activities. The study may help to shed light on the role of lectin in yam used in traditional Chinese medicine. It may also provide insights for possible therapeutic uses of the isolated nagaimo lectin.

## Materials and Methods

### Purification of Nagaimo Lectin

Nagaimo tubers were purchased from a local Japanese supermarket. The skin of nagaimo tubers was peeled off. Then the flesh was cut into small pieces of dimensions around 0.5 cm×0.5 cm. One hundred grams of the slices of nagaimo were soaked in 600 ml 10 mM Tris-HCl buffer (pH 7.6), and then homogenized in a Waring blender. The slurry was centrifuged at 30000 g, 4°C for 25 minutes. The supernatant was filtered using filter paper to yield the crude extract. Tris-HCl buffer (10 mM, pH 7.6) was added to the crude extract to adjust the volume to 1 L before loading onto an 18 cm×5 cm Q-Sepharose (GE Healthcare) column pre-equilibrated with 10 mM Tris-HCl buffer (pH 7.6). Unadsorbed materials were eluted with the starting buffer and discarded. Adsorbed materials were eluted with 1 M NaCl in 10 mM Tris-HCl buffer (pH 7.6). The fraction containing adsorbed materials was dialyzed extensively against double distilled water at 4°C, and lyophilized into powder form.

The powder was resuspended in 10 mM Tris-HCl buffer (pH 7.6) at a concentration of 15 mg/ml, and then subjected to FPLC-anion exchange chromatography on a Mono Q column (GE Healthcare) using an AKTA Purifier (GE Healthcare). Adsorbed materials were eluted using a 0–1 M NaCl gradient. The second peak containing adsorbed materials was collected, dialyzed extensively and lyophilized.

The powder was resuspended in 20 mM NaCl in 10 mM Tris-HCl buffer (pH 7.6) at a concentration of 5 mg/ml, and then subjected to FPLC-gel filtration on a Superdex 75 10/300 GL column (GE Healthcare) using an AKTA Purifier. The first major absorbance peak contained purified nagaimo lectin.

### Assay of Hemagglutinating Activity

In a 96-well microtiter U plate, a serial dilution of a 50 µl test sample was performed using phosphate-buffered saline (PBS) (pH 7.2). Then, 50 µl of a 2% rabbit red blood cell suspension in PBS was added. The plate was incubated at room temperature until the red blood cells in the blank (with no protein sample added) had fully sedimented at the bottom of the well and appeared as a red spot. Formation of plaques of agglutinated red blood cells indicated hemagglutinating activity. Specific activity of the lectin is the reciprocal of the highest dilution of the protein sample inducing hemagglutination per mg protein [22].

### Sodium Dodecyl Sulfate-polyacrylamide Gel Electrophoresis (SDS-PAGE)

Reducing SDS-PAGE involved treatment of the protein sample with loading buffer containing the reducing agent, β-mercaptoethanol, while in non-reducing SDS-PAGE, β-mercaptoethanol was not added to the loading buffer. SDS-PAGE was performed at a constant voltage of 120 V using a 15% separating gel and a 5% stacking gel. Then the gel was stained with Commassie brilliant blue for 1 hour, and destained with 10% acetic acid overnight [23].

### Molecular Mass Determination

FPLC-gel filtration was performed using a Superdex 75 10/300 GL column (GE Healthcare) previously calibrated with molecular-mass standards including phosphorylase b (97 kDa), bovine serum albumin (67 kDa), carhonic anhydrase (30 kDa), soybean trypsin inhibitor (20 kDa) and α- lactoalbumin (14.4 kDa) [24].

### N-terminal Amino Acid Sequencing

Analysis of N-terminal amino acid sequence was performed by automated Edman degradation, using a Hewlett Packard 1000A protein sequencer equipped with an HPLC system [25].

### Effects of pH, Heat and Carbohydrates on Hemagglutinating Activity of Nagaimo Lectin

In the test for pH stability, the powder of nagaimo lectin was dissolved in solutions at different pH values: pH 0–1: HCl; pH 2–5: NH_4_OAc; pH 6–10: Tris-HCl; pH 11–12: NaHCO_3;_ and pH 13–14: NaOH. After incubation at room temperature for 30 min, the solution was neutralized, and assay of hemagglutinating activity was performed as mentioned above. Percentage of residual hemagglutinating activity was calculated by dividing the hemagglutinating activity of the sample by the maximal hemagglutinating activity×100% [24].

In the heat stability test, the nagaimo lectin solution was heated at different temperatures (20°C –100°C) for 30 min. The solution was cooled on ice immediately. Then assay of hemagglutinating activity was performed [24].

In the carbohydrate test, lyophilized nagaimo lectin was dissolved in different carbohydrate solutions, all at 500 mM concentration in PBS. Assay of hemagglutinating activity was performed as mentioned above, but the carbohydrate solutions were used for serial dilution of the hemagglutinin instead of PBS. After identifying the carbohydrate specific for the lectin, a test was conducted to determine the minimal concentration of the carbohydrate for reduction of hemagglutinating activity of the lectin. The powder of nagaimo lectin was dissolved in solutions containing the specific carbohydrate at different concentrations in PBS. Assay of hemagglutinating activity was performed using the carbohydrate solutions of their particular concentrations for serial two-fold dilution instead of PBS [26].

### Assay of Anti-proliferative Activity

Suspensions of human breast cancer (MCF7), hepatoma (HepG2) and nasopharyngeal carcinoma (CNE2) cells from American Type Culture Collection were adjusted to 5×10^4^ cells/ml in RPMI medium. In a 96-well plate, 100 µl cells were seeded and incubated overnight. Then, the cells were treated with different concentrations of nagaimo lectin for 24 hours. After incubation, the medium was discarded. The wells were washed with PBS, 25 µl of 3-(4, 5-dimethylthiazol-2-yl)-2, 5-diphenyltetrazolium bromide (MTT) (5 mg/ml in PBS) were then added and further incubated for 4 hours. Then 150 µl of dimethyl sulfoxide (DMSO) were added. The absorbance at 590 nm was measured using a microplate reader within 10 min. Percentage inhibition of the cells by nagaimo lectin was calculated by: [(OD 590 nm of the control – OD 590 nm of a culture exposed to a particular lectin concentration)/OD 590 nm of the control]×100% [27].

### Flow Cytometry Analysis

About 5×10^5^ MCF7 cells were seeded on a 6-well plate overnight, and were treated with different concentrations of nagaimo lectin with or without the presence of 100 mM galactose for 24 hours. The cells were trypsinized, centrifuged down at 2000 g for 4 min, and then washed with PBS for three times. For Annexin V-FITC and PI staining, the cell pellets were resuspended in 250 µl binding buffer (0.01 M HEPES, pH 7.4, containing 140 mM NaCl and 25 mM CaCl_2_) containing 2.5 µl Annexin V-FITC (BD Phamingen, CA, USA) and 0.5 µl PI (6 mg/ml) (Sigma). The cells were incubated at room temperature in the dark for 20 min. For JC-1 staining, the cell pellets were resuspended in 500 µl plain RPMI medium containing 2.5 µg/ml JC-1, and were incubated at 37°C in the dark for 15 min. The cells were analyzed using a BD LSRFortessa cell analyzer (BD Biosciences). The signal was detected by BD LSRFortessa Blue Laser (FITC: ex 494 nm/em 519 nm, PE-Texas Red: ex 488 nm/em 615 nm, PerCP-Cy5.5: ex 482 nm/em 695 nm). Data analysis was conducted by using the program Fortessa FASDiva Program Version 6.1.3. Compensation analysis was performed by using concanavalin A as positive control for treatment of MCF7 cells [28], [29].

## Results

### Isolation of Lectin

**Figure 1:**
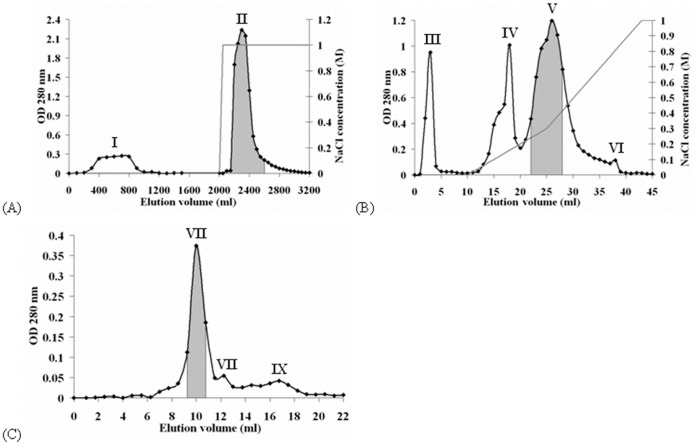
Profile of elution in purification of nagaimo lectin. Elution profile of (A) crude nagaimo tuber extract from Q-Sepharose, (B) Fraction II from Mono Q, and (C) Fraction V from Superdex 75. The shaded regions represent the fractions with hemagglutinating activity that were collected in each step.

**Figure 2:**
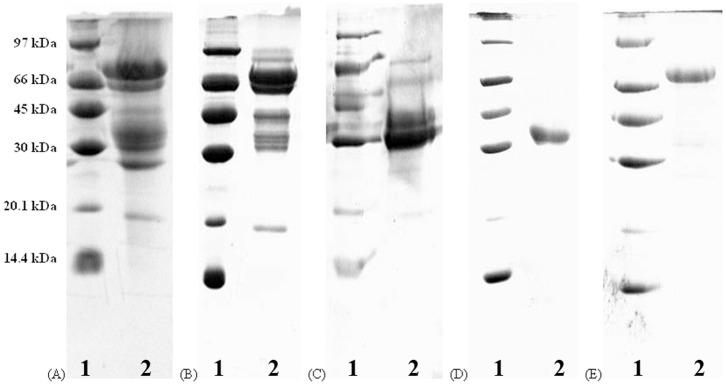
Results of SDS-APGE. SDS-PAGE of (A) crude nagaimo tuber extract, (B) Fraction II from Q-Sepharose, (C) Fraction V from Mono Q, (D) Purified nagaimo lectin from Superdex 75, and (E) Purified nagaimo lectin without addition of the reducing agent, β-mercaptoethanol. Lane 1 of each panel: Molecular weight markers (GE Healthcare) including phosphorylase b (97 kDa), bovine serum albumin (66 kDa), ovalbumin (45 kDa), carbonic anhydrase (30 kDa), soybean trypsin inhibitor (20 kDa) and α-lactalbumin (14.4 kDa). Lane 2 of each panel: nagaimo protein samples.

A protocol with three chromatographic steps was used in lectin purification from nagaimo tubers. The first step, cation exchange chromatography on Q-Sepharose, yielded an unabsorbed fraction (Fraction I in Fig. 1A) and a sharp adsorbed fraction (Fraction II in Fig. 1A). The latter fraction was collected and subjected to the second step, FPLC-cation exchange chromatography on Mono Q. This step yielded one unadsorbed fraction (Fraction III in Fig. 1B), and two major adsorbed peaks (Fraction IV and V in Fig. 1B) and one minor adsorbed peak (Fraction VI in Fig. 1B). Hemagglutinating activity resided in Fraction V. This fraction was subjected to the third step, FPLC-gel filtration on Superdex 75. The first, major peak (Fraction VII in Fig. 1C) showed hemagglutinating activity but not the two minor peaks (Fraction VIII and IX in Fig. 1C). Fraction VII contained purified nagaimo lectin that appeared as a single 35-kDa band in SDS-PAGE (Fig. 2). The protocol contributed to purification of nagaimo lectin by approximately 33.4 folds (Table 1).

**Table 1:** Table of purification of lectin from nagaimo tubers (100 grams).

Step of purification	Yield (mg)	Specific hemagglutinatingactivity (unit)	Total hemagglutinatingactivity (10^6^ units)	Recovery of activity (%)	Fold of purification
Extract	12200	1024	12.49	100	1
Q-Sepharose	979.3	10774	10.55	84.46	10.52
Mono Q	314.0	24576	7.72	61.76	24.00
Superdex 75	170.8	34198	5.84	46.76	33.40

In non-reducing SDS-PAGE, without treatment with β-mercaptoethanol, the nagaimo lectin appeared as a single 70-kDa band instead (Fig. 2). In FPLC-gel filtration on a Superdex 75 10/300 GL column, nagaimo lectin was eluted in the 10^th^ ml. Based on the calibration curve for the column, the molecular mass of nagaimo lectin was 70-kDa. This showed that nagaimo lectin is a 70-kDa dimeric protein with two 35-kDa subunits.

### N-terminal Amino Acid Sequence

The first 12 amino acids at the N-terminus of nagaimo lectin were NPFVFFVAINNP. Protein BLAST was applied to seek homologous proteins in the GenBank Database. The proteins with highest scores are listed in Table 2. They are highly different proteins with different sizes and present in different origins. However, none of them seem to be related to lectins. The N-terminal amino acid sequence of nagaimo lectin could not match any plant lectins, nor any types of *Dioscorea* proteins.

**Table 2:** N-terminal amino acid sequence of nagaimo lectin, and the amino acid sequences of other proteins with highest homology based on the GenBank Database.

Identity	Accession number	N-terminal sequence	% identity
Nagaimo lectin [*Dioscorea opposita*]	–	^1^NPFVFFVAINNP^12^	100
Omp121 family outer membrane protein [*Flavobacterium psychrophilum* JIP02/86]	CAL43282.1	^987^NPFVFFVDKNNF ^998^	75
ssDNA-binding protein [*Mycoplasma leachii* 99/014/6]	YP_005907746	^23^NPFVFFTVAVNEY ^35^	66.7
methylated-DNA–protein-cysteine methyltransferase [*Hippea maritima*]	YP_004339567	^114^NPFVFFVACHRV ^125^	66.7
acetyl-CoA hydrolase/transferase [*Loa loa*]	XP_003147030	^296^NPFVFFGDVAWVNDP^310^	66.7
ABC transporter ATP binding protein [*Escherichia coli*]	ABY84911	^54^ EAFVFIVDINNP^65^	66.7

The amino acids in other proteins differ from that of nagaimo lectin are underlined.

### Thermostability and pH Stability

**Figure 3:**
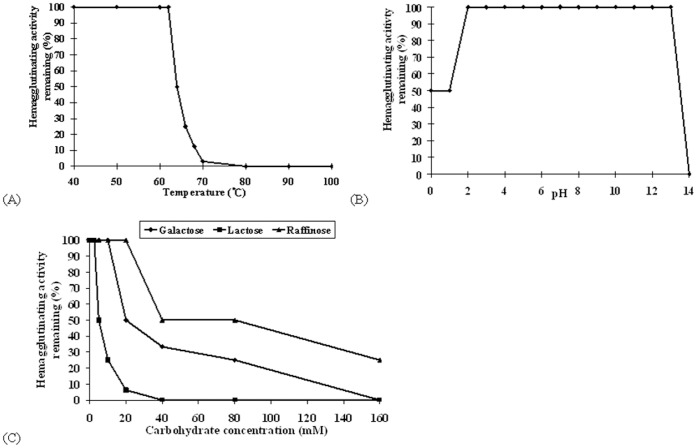
Effect of (A) different temperatures, (B) different pH, and (C) specific carbohydrates on hemagglutinating activity of Nagaimo lectin.

Nagaimo lectin had moderate thermostability. It retained full hemagglutinating activity up to 62°C, but the activity dropped abruptly when the temperature was elevated from 64°C to 70°C, and vanished at 80°C (Fig. 3A). On the other hand, nagaimo lectin showed fairly high pH stability. Hemagglutinating activity was preserved at pH 2 to 13, while the activity was halved at pH 0–1 and totally eliminated at pH 14 (Fig. 3B).

### Carbohydrate Specificity

In the carbohydrate specificity test of nagaimo lectin, the lectin did not interact with glucose, mannose, glucosamine, glucuronic acid, galactonic acid, xylose, xylitol, mannitol and arabinose. In addition of galactose, nagaimo lectin also interacted with galactose-containing carbohydrates including lactose (glucose+galactose) and raffinose (glucose+fructose+galactose), as indicated by reduction in hemagglutinating activity of the lectin (Fig. 3C). The minimal concentration of galactose, lactose and raffinose required for reduction of nagaimo lectin hemagglutinating activity was 20 mM, 5 mM and 40 mM, respectively. The inhibitory effect of lactose on nagaimo lectin was the strongest, indicating the lectin has a high tendency to interact with β-galactosides. The carbohydrate specificity of nagaimo lectin was not limited to β-galactosides, since it also interacted with α-galactosides (raffinose) and galactose alone.

### Anti-proliferative Activity Toward MCF7, HepG2 and CNE2 Cells

**Figure 4:**
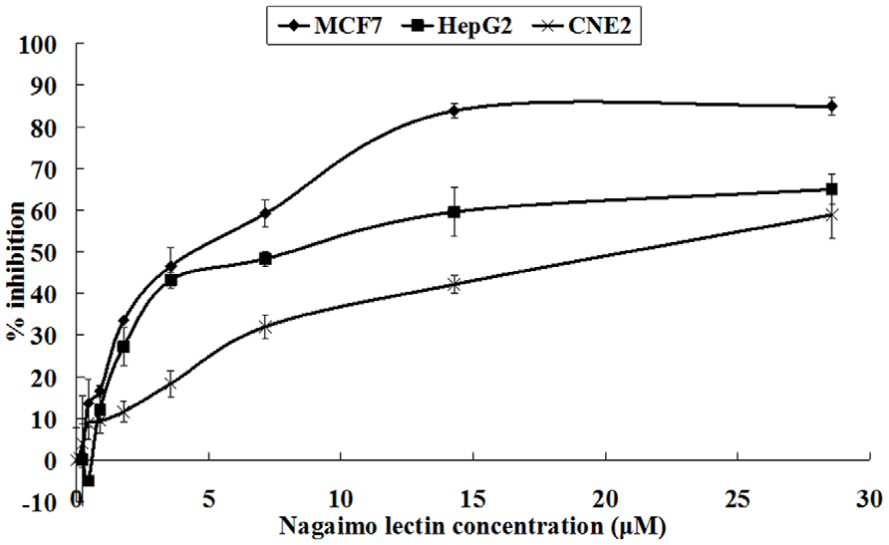
Results of MTT assay on different cell lines. Results of MTT assay of MCF7, HepG2 and CNE2 cells treated with different concentrations of nagaimo lectin. Results represent mean±SD (n = 3).

Nagaimo lectin also exhibited anti-proliferative activity. Results of the MTT assay disclosed that treatment with nagaimo lectin for 24 hours strongly inhibited the growth of MCF7 and HepG2 cells, with IC_50_ of 3.71 µM and 7.12 µM, respectively. It also slightly inhibited growth of CNE2 cells, with an IC_50_ of 19.79 µM (Fig. 4).

**Figure 5:**
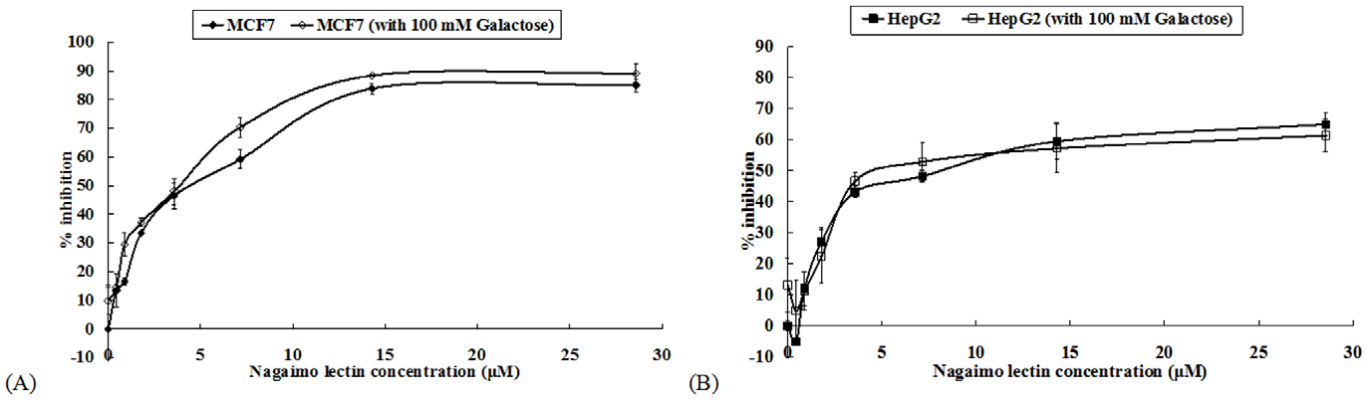
Results of MTT assay of (A) MCF7 cells and (B) HepG2 cells treated with nagaimo lectin, with or without concurrent administration of 100 mM galactose. The presence of 100 mM galactose could not curtail the retarding effect of nagaimo lectin on the growth of MCF7 and HepG2 cells. Results represent mean±SD (n = 3).

Co-treatment with 100 mM galactose, the specific binding sugar of nagaimo lectin, failed to diminish the anti-proliferative activity of nagaimo lectin on the MCF7 and HepG2 cells (Fig. 5). In the test of inhibitory effects of carbohydrates on hemagglutinating activity of nagaimo lectin, 100 mM galactose could lower the activity by about 80%, however, such inhibitory action of galactose was not observed on the anti-proliferative activity of nagaimo lectin.

**Figure 6:**
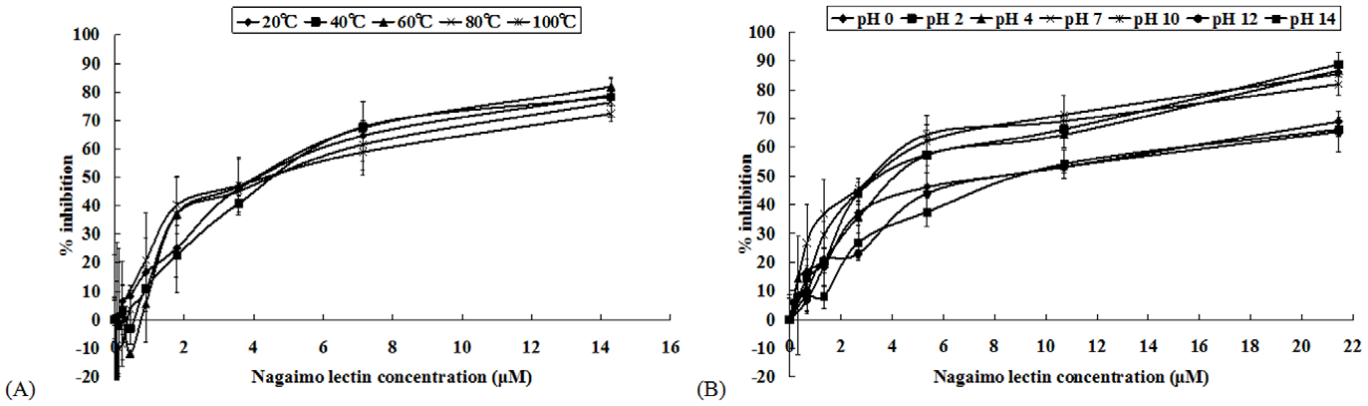
Results of MTT assay of MCF7 cells treated with nagaimo lectin that were pre-treated at different (A) temperatures and (B) pH values for 30 minutes. The presence of 100 mM galactose could not curtail the retarding effect of Nagaimo lectin on the growth of MCF7 and HepG2 cells. Results represent mean±SD (n = 3).

Similarly, heat treatment on nagaimo lectin failed to abolish its anti-proliferative activity on the MCF7 cells (Fig. 6A). Unlike the hemagglutinating activity which had diminished since 64°C and vanished at 80°C, full anti-proliferative activity of nagaimo lectin was retained even the lectin was heated at 80°C and 100°C for 30 minutes.

On the other hand, anti-proliferative activity of nagaimo lectin on MCF7 cells had declined after extreme pH treatments (Fig. 6B). The activity was not affected at pH 2, 4 and 10. At pH 0, 12 and 14, the IC_50_ on MCF7 cells had raised by over 110% to around 8 µM, indicating reduction of anti-proliferative activity of nagaimo lectin. Hemagglutinating activity of nagaimo lectin was stable at pH 2 to 13. Anti-proliferative activity of nagaimo lectin was more vulnerable in alkaline pH, as observed by activity loss at pH 12.

These suggested that nagaimo lectin interacted with the cancer cells through a domain other than the sugar binding site of the lectin. The blockage of sugar binding sites by galactose did not hinder the interaction between the domain on nagaimo lectin and the cancer cells, with the consequence that the anti-proliferative activity remained intact.

### Induction of Apoptosis in MCF7 Cells

**Figure 7:**
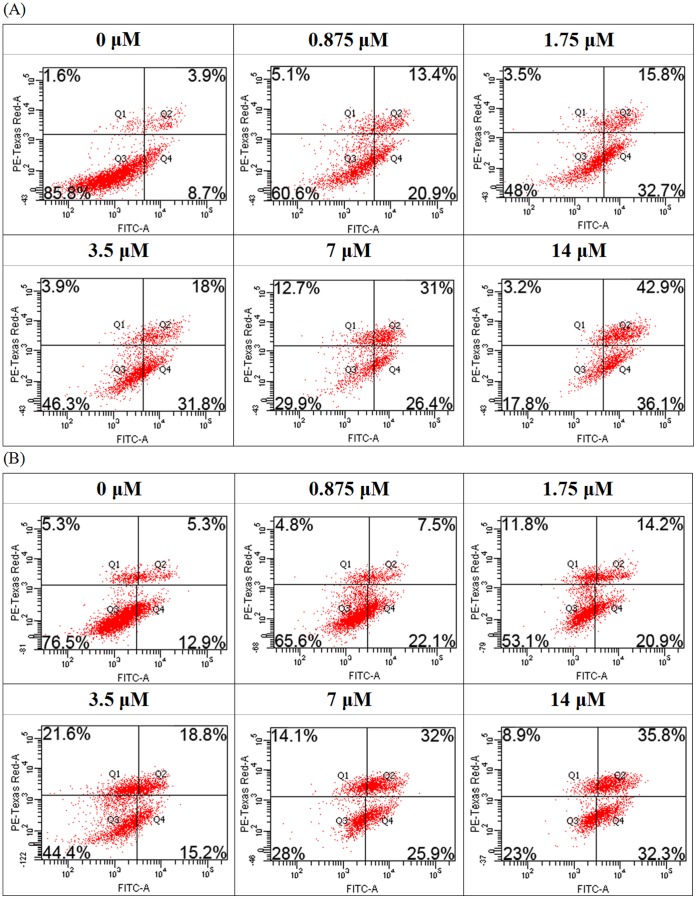
Flow cytometry analysis on Annexin V-FITC and PI staining. Flow cytometry analysis of MCF7 cells after treatment with nagaimo lectin (A) with or (B) without the presence of 100 mM nagaimo lectin for 24 hours, followed by staining with Annexin V-FITC.

Flow cytometry analysis was used for further studies on nagaimo lectin-induced anti-proliferative effects on MCF7 cells. After Annexin V-FITC and propidium iodide (PI) staining, the MCF7 cells at the lower left quadrant of the profile shifted toward the lower right quadrant as nagaimo lectin concentration increased (Fig. 7A). The shifting indicated phosphatidylserine (PS) externalization in the MCF7 cells which were undergoing the early stage of apoptosis. The proportion of cells located at the upper right quadrant had also increased slightly at increasing nagaimo lectin concentrations, justifying that more MCF7 cells entered the late stage of apoptosis.

**Figure 8:**
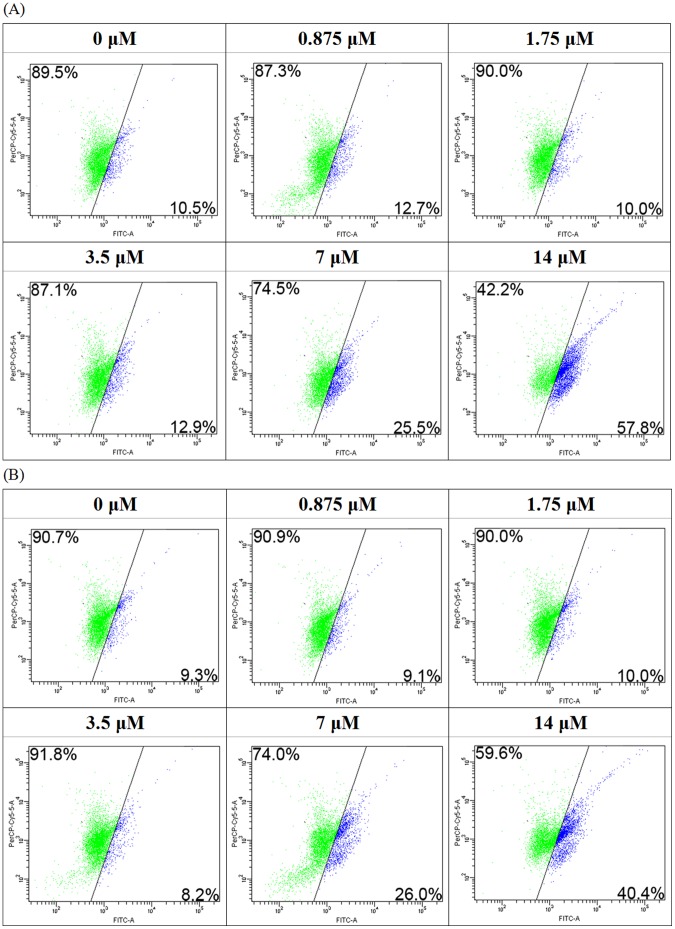
Flow cytometry analysis on JC-1 staining. Flow cytometry analysis of MCF7 cells after treatment with nagaimo lectin (A) with or (B) without the presence of 100 mM nagaimo lectin for 24 hours, followed by staining with JC-1.

In JC-1 staining, a slight shift of MCF7 cells from the upper left region toward the lower right region was observed (Fig. 8A). The cell shifting showed an increase in the proportion of MCF7 cells experiencing mitochondrial depolarization and undergoing cell death.

With the presence of 100 mM galactose, nagaimo lectin still could induce PS externalization and mitochondrial depolarization in the MCF7 cells (Fig. 7B, Fig. 8B). Occupation of carbohydrate binding sites of nagaimo lectin could not diminish the pro-apoptotic effect of the lectin on MCF7 cells.

## Discussion

Lectins have been identified in various *Dioscorea* species, including *D. alata, D. cayenesis, D. polygonoides, D. rotundata* [30], [31] and *D. batatas* [32]. However, very few of them have been investigated in great detail. *D. batatas* lectins were the best studied *Dioscorea* lectins. *D. batatas* produced three lectins with distinct characteristics, DB1, DB2 and DB3. DB1 was a 20-kDa mannose-binding insecticidal lectin with two 10-kDa subunits. DB2 was a 31-kDa maltose binding lectin which served as the major storage protein in *D. batatas*. DB3 was a 128-kDa maltose binding lectin compsed of a 66-kDa subunit and two 31-kDa subunits [32], [33].

Studies on the three lectins from *D. batatas* were comprehensive [32], [33]. The isolation of multiple lectins was elegantly performed. Differences in the molecular size, amino acid sequence, heat and pH stability, and sugar binding specificity among the three lectins have been demonstrated. The variations in the composition of the three lectins also helped to illustrate the physiological roles of these lectins in the yam tubers. DB2 had N-terminal amino acid sequence resembling *Dioscorea* dioscorins that act as yam storage proteins. However, unlike dioscorins, DB2 did not exhibit carbonic anhydrase activity [32].

In contrast, the lectin that we purified from nagaimo (*D. opposita*) did not resemble any of aforementioned *Dioscorea* lectins. In contrast to the multiplicity of lectins in *D. batatas* [32], [33], nagaimo yielded only a single 70-kDa lectin. None of the other chromatographic fractions acquired during the course of nagaimo lectin purification manifested hemagglutinating activity. This suggested that no other lectin was present in nagaimo tubers except nagaimo lectin. DB2 in *D. batatas* acted as the major storage protein in the tubers, but not nagaimo lectin in nagaimo tubers. SDS-PAGE of the crude extract of nagaimo tubers revealed that the 35-kDa band representing the lectin was only one of the major protein bands. It deserves mention that some *Phaseolus* species and *Phaseolus vulgaris* cultivars have only a solitary defense protein [34]–[37] whereas other *Phaseolus* species and *Phaseolus vulgaris* cultivars produce two or more defense proteins [38]–[39].

The N-terminal amino acid sequence of nagaimo lectin was NPFVFFVAINNP. The sequence did not resemble any of the lectins in *D. batatas* [32]. Structurally disparate straw mushroom (*Volvariella volvacea*) lectins have been reported from different research laboratories [40], [41]. Also, the sequence of nagaimo lectin did not show homology to the *Dioscorea* dioscorins found in the GenBank Database. Dioscorins are one of the major storage proteins in yams. With no resemblance in sequence, it seems that nagaimo lectin is another storage protein in *D. opposita* tubers other than dioscorins. The results from the Protein BLAST search in GenBank Database even showed that the N-terminal sequence of nagaimo lectin did not resemble the sequence of any plant lectins. The most homologous sequence from the database was an Omp121 family outer membrane protein from *Flavobacterium psychrophilum*, a gram-negative bacteria, with around 75% identity within the sequence (Table 2). However, it has a much larger size (1060 amino acids), and the sequence is located near the C-terminus (987^th^ to 997^th^ amino acids) instead. It is unlikely to be related to nagaimo lectin. Nagaimo lectin is probably a new plant lectin with a distinct N-terminal amino acid sequence.

Nagaimo lectin displayed higher thermostability and pH stability than *D. batatas* lectins [32], [33]. Nagaimo lectin was heat-stable up to 62°C, while DB1 and DB3 started to lose hemagglutinating activity after heating at 50°C. Nagaimo lectin was stable over a wider pH range (pH 2–13) than DB1 (pH 7–9) and DB3 (pH 3–9). Besides, the carbohydrate binding specificity was also distinctly different. Nagaimo lectin was specific to galactosides and also interacted with galactose, while the lectins from *D. opposita* were mannose- and maltose-specific. Even though they belong to the same genus, they produce totally dissimilar lectins.

The investigations on biological activities of lectins from *D.* *batatas* were focused on their insecticidal activity [33], A study on anti-tumor activity was not included. Also, none of the other *Dioscorea* lectins have been reported to have anti-proliferative activity. The present study constituted the first report of a *Dioscorea* lectin possessing anti-proliferative activity on tumor cells. Nagaimo lectin from *D. opposita* exhibited anti-proliferative activity on several types of tumor cells, comprising breast cancer MCF7 cells, hepatoma HepG2 cells and nasopharyngeal carcinoma CNE2 cells. The inhibitory activity on MCF7 and HepG2 cells was more potent than that on CNE2, with over 2.5-fold difference in IC_50_ values (IC_50_ of MCF7 cells: 3.71 µM, HepG2 cells: 7.12 µM, CNE2 cells: 19.79 µM). The ability of nagaimo lectin to potently interfere with the proliferation of several types of cancer cells is noteworthy in view of the reports that some lectins/hemagglutinins are devoid of anti-proliferative activity [37]. Flow cytometry analysis of nagaimo lectin-treated MCF7 cells revealed that the lectin induced apoptosis which brought about phosphatidylserine externalization and mitochondrial depolarization.

Many reports have shown that lectins induce apoptosis in tumor cells through their carbohydrate binding capability [42]–[47]. For example, artinM, a d-mannose-binding lectin from jackfruit, bound onto and induces apoptosis in myeloid leukemia NB_4_ cells, and the activity was abolished in the presence of Manα1-3[Manα1-6]Man [45]. In contrast, nagaimo lectin seemed to make use of other domains instead of its galactose-binding domain to interact with tumor cells. The application of galactose could not suppress the anti-proliferative effect of nagaimo lectin on tumor cells. This observation is reminiscent of an analogous finding regarding the glucose-mannose binding *Canavalia gladiata* lectin. Its anti-proliferative activity was not attenuated in the presence of glucose [48].

Unlike the hemagglutinating activity of nagaimo lectin, the anti-proliferative activity was not affected by galactose. Similarly, heating could not destroy the anti-proliferative activity of nagaimo lectin but the hemagglutinating activity. On the other hand, the anti-proliferative activity of nagaimo lectin was more vulnerable to alkaline pHs than the hemagglutinating activity. All these observations support that, two distinct domains are responsible for the two kinds of activity, i.e. the carbohydrate binding domain is independent of the anti-proliferative activity. Not limited to inhibition of MCF7 cell proliferation, nagaimo lectin also induced apoptosis on them. This was indicated by the ability of nagaimo lectin to induce PS externalization and mitochondrial depolarization on the cells. Same as the anti-proliferative activity, the apoptosis-inducing effects of nagaimo lectin was not suppressed by galactose. The initiation of apoptosis was also independent of the carbohydrate binding capacity of the lectin.

The hemagglutinating activity of nagaimo lectin was attenuated by galactosides and also galactose. The hemagglutinating activity of lectins is one of the major obstacles in the application of lectins for therapeutic purposes. A number of lectins exhibit anti-tumor and hemagglutinating activities at the same time through their carbohydrate binding activity [46], [47]. The hemagglutinating activity of lectins would constitute a side effect that leads to red blood cell agglutination in patients when applying lectins for cancer treatment. Nagaimo lectin probably did not depend on its carbohydrate binding capability to induce apoptosis in tumor cells. It is possible to introduce galactose and galactose-containing sugars to block the hemagglutinating effect of the lectin, while not hindering its anti-tumor activity. This suggests that nagaimo lectin has the potential to be the first *Dioscorea* lectin in therapeutic application.

Some [36], [49], [50] but not other [34]–[37], [48] lectins demonstrate antifungal activity. Nagaimo lectin is destitute of antifungal activity. In fact, antifungal and trypsin inhibitory activities which are found in the tubers of other species, are lacking in nagaimo tubers (data not shown).

This report adds to the meager literature on bioactive proteins in the yam tuber. Nagaimo lectin isolated in the present investigation is distinctive in certain aspects. It possesses a molecular mass, N-terminal sequence, sugar specificity, pH stability and thermostability distinct from previously reported yam lectins. It exhibits potent anti-proliferative activity toward several cancer cell lines.

Information pertaining to the ability of constituents of tubers, rhizomes and seeds, especially proteinaceous components, to inhibit the growth of breast cancer cells, is meager [51]–[55]. The compound dioscorealide B from *Dioscorea membranacea* is active against breast cancer cells [56]. Contamination of nagaimo lectin with small molecules like dioscorealide B is highly unlikely due to the vast difference in their molecular masses and also the use of extensive dialysis between the chromatographic steps.

Breast cancer is one of the most prevalent and severe gynecologic diseases [57]. Globally, the incidence was 0.641 million cases in 1980 and it soared to 1.643 million cases in 2010 [58]. There were 425 000 women who died of breast cancer in 2010 [28]. Nagaimo lectin is potentially exploitable for the treatment of this formidable disease.
